# A case report of BCR-ABL(+) acute myeloid leukemia and literature review

**DOI:** 10.3389/fonc.2026.1772154

**Published:** 2026-04-14

**Authors:** Jianping Li, Wei Zheng, Xueyan Dong, Jingyi Wang, Kui Liu

**Affiliations:** Department of Hematology, Affiliated Hospital of Shandong University of Traditional Chinese Medicine, Jinan, Shandong, China

**Keywords:** allogeneic hematopoietic stem cell transplantation, BCR-ABL(+) acute myeloid leukemia, prognosis, treatment, tyrosine kinase inhibitors

## Abstract

BCR-ABL(+) acute myeloid leukemia (AML) is considered a rare AML subtype with a short median survival time and poor prognosis. Currently, there is no unified standard treatment regimen for this disease, which complicates clinical diagnosis, treatment and medication selection. Case reports on this AML subtype remain relatively scarce to date. In this study, we present a case of BCR-ABL(+) AML that was treated with a TKI in combination with chemotherapy to induce remission, followed by allogeneic hematopoietic stem cell transplantation (Allo-HSCT). We also review the relevant literature to shed light on treatment strategies for BCR-ABL(+) AML and provide strong support for Allo-HSCT as a therapeutic option for this subtype.

## 1 Introduction

Acute myeloid leukemia (AML) is a highly heterogeneous hematological malignancy that arises from the malignant proliferation of hematopoietic stem/progenitor cells. It can result from various genetic alterations that disrupt cellular differentiation and maturation, as well as inhibiting apoptosis. This leads to the accumulation of leukemic stem cells. The BCR-ABL fusion gene is a hallmark of chronic myeloid leukemia (CML) and involves translocations between chromosomes 9q34 and 22q11, also known as the Philadelphia chromosome (Ph). Reports indicate that the BCR-ABL fusion gene is present in all CML patients, 20–30% of adult acute lymphoblastic leukemia (ALL) cases, 2–5% of pediatric ALL cases and 0.5–3% of acute myeloid leukemia (AML) patients (1). Therefore, a clinical diagnosis of this disease requires the exclusion of CML and MPAL. Key exclusion criteria include the absence of a prior history of CML, a low basophil percentage in peripheral blood/bone marrow and an absence of significant splenomegaly. Further differentiation necessitates immunophenotyping, genetic analysis and cytogenetic testing.

In leukemia cells, the BCR-ABL fusion gene disrupts cellular signaling pathways and destabilizes genomic integrity. It persistently enhances tyrosine kinase (TK) activity, and these abnormally activated kinases interfere with downstream signaling pathways. This leads to increased cell proliferation, differentiation arrest, and resistance to cell death (2). BCR-ABL(+) AML patients exhibit low complete remission (CR) rates, high relapse risks, and a median survival of approximately nine months, indicating an extremely poor prognosis. Currently, there is no unified treatment protocol (3). Thus, the BCR-ABL fusion gene plays a pivotal role in diagnosing leukemia and determining prognosis. To explore the clinical features, diagnosis, treatment, and prognosis of BCR-ABL(+) AML, this paper presents a case study and reviews relevant literature. The aim is to deepen the understanding of this disease category and thereby improve treatment outcomes.

## 2 Case data

Patient Liu, a 48-year-old male, was admitted with the chief complaint of abnormal blood counts discovered over 10 days ago. At the time of admission, he presented with: He was alert and oriented. He had no fever, dizziness, palpitations, nausea, vomiting, or abdominal discomfort. He had a fair appetite and sleep, and normal bowel and bladder function. Physical examination revealed no positive findings. His past medical history included a diagnosis of “malignant tumor of the chest wall” over ten years prior. Pathology results from a chest wall biopsy showed predominantly mature skeletal muscle with inflammatory tissue and microscopic tissue proliferation. Focal areas with significant cellular atypia were identified, suggesting malignancy. Immunohistochemistry indicated the possibility of chondrosarcoma, liposarcoma, or malignant fibrous histiocytoma. Immunohistochemistry: S100 focal (+/–), CD34 (–), desmin (–), CK (–), CD68 focal (+), Ki67 scattered (+). The patient previously underwent radiotherapy and chemotherapy. There is a history of hepatitis B virus carrier status.

Upon admission, the following auxiliary tests were completed: Complete Blood Count: White blood cells: 47.49×10^9^/L,Monocytes: 18.87×10^9^/L,Lymphocytes: 24.53×10^9^/L,Eosinophils: 0.66×10^9^/L,Basophils: 0.17×10^9^/L,Hemoglobin: 114 g/L,Platelets: 73×10^9^/L. Abnormal leukocyte morphology: 58% blast cells. Infectious Series: Hepatitis B surface antigen (+); e antibody (+); core antibody (+); Hepatitis B DNA quantitative: <500. LDH: 348 U/L. Bone marrow cytomorphology: Extremely hyperplastic bone marrow with 57% visible blast cells. Morphology is consistent with acute myeloid leukemia (AML). Immunophenotyping: Abnormal cell populations extending from the blast to the myeloid cell regions constitute approximately 55.5% of nucleated cells. The cells expressed HLA-DR, CD13, CD34, and CD38, and some expressed CD19 and CD117. Granulocytes showed abnormal differentiation with CD16-CD13 and CD11b-CD13 dot-blot patterns. Conclusion: Acute myeloid leukemia (AML-M2 or M4 possible). WT1: Copy number: 17,490; WT1/ABL ratio: 8.786%; high expression.BCR-ABL fusion gene: P190 type. Positive (+); BCR-ABL/ABL ratio: 166.436%. Myeloid 248 gene mutations: ASXL1 (43.58%) and RUNX1 (88.77%). Karyotype: 46, XY, t(9;22)(q34;q11) [20]. Bone marrow biopsy: Consider acute myeloid leukemia with partial CD19 expression. Confirmed diagnosis: Acute myeloid leukemia (P190+, ASXL1+, RUNX1+, poor prognosis).

On April 15, 2024, the patient received HA plus imatinib (imatinib 400mg qd; high-dose vinorelbine: 2mg d1-7; Cytarabine: 0.2g d1-7). On day 28 of treatment, follow-up bone marrow cytomorphology showed CR, flow cytometry indicated MRD-negative status, and BCR-ABL/ABL ratio was 14.216%. On May 16, 2024, treatment with Imatinib + HAG regimen was administered (Imatinib 400mg qd; Granulocyte Colony-Stimulating Factor: 300μg d0-7; Homoharringtonine: 2mg d1-7; Cytarabine: 0.2 g d1-7). Post-treatment bone marrow review indicated CR, MRD negative, BCR-ABL/ABL: 0.73%.

On June 17, 2024, and July 18, 2024, the patient underwent consolidation therapy with imatinib plus high-dose cytarabine (HD-Ara-C) (Imatinib 400mg qd; HD-Ara-C: 6.6g q12h qod). During this period, bone marrow aspiration indicated complete remission (CR). Lumbar puncture and intrathecal injection were performed to prevent central nervous system infiltration; cerebrospinal fluid analysis revealed no abnormalities. but BCR-ABL remained detectable. After informing the patient and family about the condition and risks associated with transplantation, allogeneic hematopoietic stem cell transplantation (Allo-HSCT) was planned.

Patient 2024–8 presented with pain in the right lower quadrant of the abdomen. A color Doppler ultrasound revealed nodular enlargement of the mid-appendiceal segment with an occupying lesion that could not be excluded. A PET-CT scan revealed a soft tissue mass in the appendix region with increased FDG metabolism, which suggests extramedullary leukemia infiltration. Cefoperazone/sulbactam was administered to control the infection, followed by a laparoscopic appendectomy. Pathology results showed chronic inflammatory changes in the appendix. The patient developed postherpetic neuralgia and acute hepatitis B virus flare postoperatively, adding insult to injury. Antiviral therapy was initiated and imatinib was maintained throughout. On September 24, 2024, treatment commenced with Azacitidine (154 mg, days 1–7) and Imatinib 400 mg once daily.

10/16/2024 Bone marrow morphology: CR, MRD negative. BCR-ABL fusion gene: P190(+), 0.056%; RUNX1: 0.006%. The patient underwent Allo-HSCT in November 2024. The donor was the patient’s 22-year-old son and was HLA-matched as 5/10 hemichip. Preconditioning regimen: BU/CY + ATG protocol: Busulfan (0.8 mg/kg every six hours on days -10, -9, and -8), semustine (250 mg on day -7), cytarabine (8 g/m² on days -6 and -5), cyclophosphamide (100 mg/kg on days -4 and -3), and ATG (100 mg on day -5, 150 mg on days -4 and -3, and 200 mg on day -2). The child received approximately 260 mL of peripheral blood from the father with the following counts: The patient weighed 80.4 kg, and the calculated MNC was 5.88 × 10^8^/kg and the calculated CD34+ was 4.77 × 10^6^/kg. Cyclosporine, mycophenolate mofetil, and methotrexate were administered for graft-versus-host disease prophylaxis. Neutrophils engrafted on day +16 and platelets on day +21.

**Figure 1:**
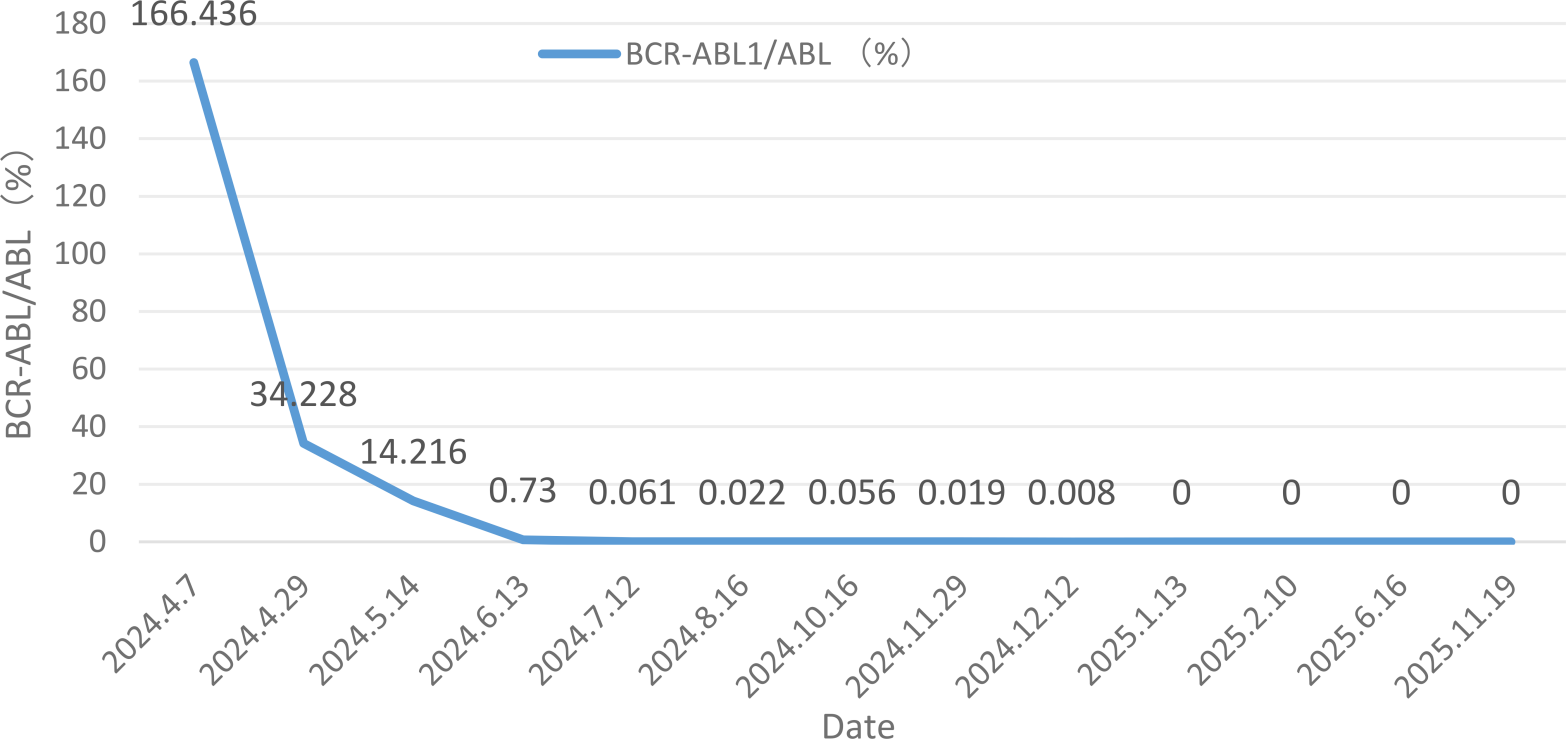
BCR-ABL/ABL before and after treatment.

**Figure 2:**
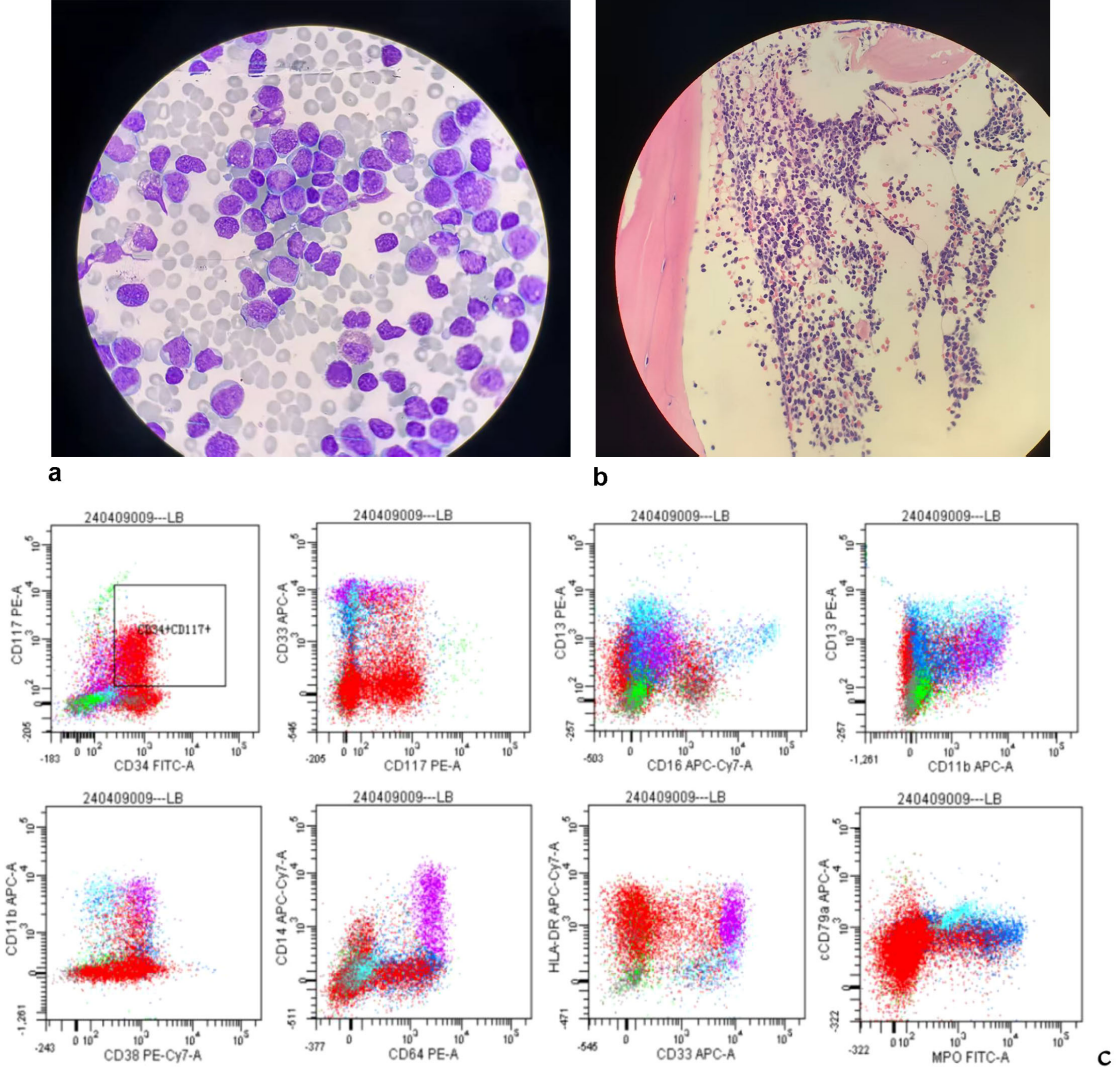
**(a)** Riether-Gimsa staining, bone marrow cell morphology in BCR-ABL(+) AML patients; **(b)** Bone marrow pathology in BCR-ABL(+) AML patients; **(c)** Immunophenotyping in BCR-ABL(+) AML patients.

The patient maintained complete remission (CR) of bone marrow morphology at +1, +2, +3, +6 and +12months post-transplant. Minimal residual disease (MRD) remained negative, BCR-ABL turned negative, and the chromosomal karyotype reverted to normal. Chimeric analysis revealed a 100% chimerism rate. To date, the patient remains in remission with no evidence of graft-versus-host disease (GVHD). The patient is undergoing maintenance therapy with imatinib 400 mg. **Table 1** summarizes the patient’s pre-transplant treatment course, **Table 2** details the pre-transplant bone marrow results, and **Figure 1** illustrates the BCR-ABL/ABL levels before and after treatment. The patient's bone marrow cytomorphology, bone marrow biopsy, and immunophenotyping results are shown in **Figure 2**.

**Table 1:** Patient treatment process prior to transplantation.

Date	Treatment plan
2024-4-15	imatinib(400mg)+HA(H:2mg d1-7, Arc:0.2g d1-7)
2024-5-16	imatinib(400mg)+HAG(G-CSF:300ug d0-7, H:2mg d1-7, Arc:0.2g d1-7)
2024-6-17	imatinib(400mg)+HD-ARAC(6.6g q12h qod )
2024-7-18	imatinib(400mg)+HD-ARAC(6.6g q12h qod )
2024-9-24	imatinib(400mg)+AZA(154mg, d1-7)

**Table 2:** Bone marrow results of patients before and after transplantation.

Date	Bone marrow cell morphology	MRD	BCR-ABL/ABL	ASXL 1	RUNX 1
2024.4.7	58% blast cells		166.436%	43.58%	88.77%
2024.4.29	CR	1.15%	34.228%	—	—
2024.5.14	CR	negative	14,216%	—	—
2024.6.13	CR	negative	0.73%	negative	0.27%
2024.7.12	CR	negative	0.061%	—	0.06%
2024.8.16	CR	negative	0.022%	—	0.09%
2024.10.16	CR	negative	0.056%	negative	0.06%
2024.11.29	CR	negative	0.019%	—	—
2024.12.12	CR	negative	0.008%	negative	negative
2025.2.10	CR	negative	negative	—	—

## 3 Literature review and discussion

BCR-ABL(+) AML is considered a distinct, rare subtype. While the 2016 revised WHO guidelines classified it as a provisional category of AML with recurrent genetic abnormalities, the 2022 WHO guidelines designated it as an independent subtype. Unlike other genetically defined AML subtypes, diagnosis requires the presence of the BCR-ABL fusion gene in ≥20% of blasts to differentiate it from CML-AP (4).Patients with BCR-ABL(+) AML have an extremely poor prognosis, with a median survival of only 7–9 months. Most reports indicate that patients with BCR-ABL(+) AML exhibit early relapse after standard chemotherapy regimens. This led the 2017 European Leukemia Network to classify them as a high-risk group. Initial studies by the UK Medical Research Council revealed that the five-year overall survival rate for 47 BCR-ABL(+) AML patients was just 11% (5, 6).Studies such as that by Mizuno et al. analyzed transplant outcomes for BCR-ABL(+)AML patients, comparing them with those with intermediate-risk cytogenetics and no Ph abnormalities. The results showed that Ph(+) patients had OS, DFS and relapse rates similar to those with intermediate-risk AML. However, the relapse rate after allo-HSCT was significantly reduced (7).

The diagnosis of BCR-ABL(+) AML currently requires the exclusion of CML. Compared to CML, BCR-ABL(+) AML is clinically characterized by less pronounced splenomegaly, fewer peripheral basophils and a lower myeloid-to-erythroid cell ratio in the bone marrow (3).Research reports indicate that CML almost exclusively carries BCR-ABL1 major breakpoint (M-bcr) transcripts (b2a2 or b3a2, which encode the p210 protein), whereas BCR-ABL(+) AML may express minor breakpoint (m-bcr) transcripts (e1a2, which encode p190) or rare u-bcr variants (8).In molecular biology, most CML patients harbor mutations in the RUNX1 and ASXL1 genes. These are followed by mutations in chromatin and spliceosome genes, such as BCORL1, BCOR, PHF6 and U2AF1, as well as mutations in IDH1/2, NRAS, TET2 and TP53 (9, 10); A retrospective study of BCR-ABL(+) AML identified RUNX1 as the most commonly mutated gene, present in around 38% of cases, compared to 10% in *de novo* AML. Relevant mutations were also found in genes involved in RNA splicing (SRSF2 and SF3B1) and chromatin regulation (ASXL1, STAG2, BCOR and BCORL1). However, mutations were not detected in genes commonly associated with AML, such as NPM1, FLT3 or DNMT3A (11).Similar findings were observed in studies by Eisfeld et al., in which BCR-ABL(+) AML patients exhibited high mutation rates in RUNX1, as well as in genes associated with chromatin regulation and RNA splicing (9).Therefore, the high prevalence of RUNX1 mutations, coupled with the absence of NPM1 and FLT3 mutations, does not appear to constitute a defining characteristic of BCR-ABL(+) AML.

Additionally, cytogenetic abnormalities are more prevalent in CML, manifesting as +8, +Ph and 3q26.2 rearrangements. In contrast, the most common abnormalities in BCR-ABL(+) AML patients are -7, +8 and complex karyotypes (12, 13).Research has found that, compared to CML, BCR-ABL(+) AML exhibits a lower incidence of cytogenetic abnormalities, a higher incidence of +19, and a similar incidence of +8. Furthermore, the findings suggest that the presence of multiple genetic abnormalities is often associated with a relatively unfavorable prognosis (8).Other studies have reported that restoration of a normal karyotype after induction chemotherapy is more common in patients with BCR-ABL(+) AML, whereas the Philadelphia chromosome persists in CML patients even during remission (8).Recent genomic data reveal that BCR-ABL(+) AML shares another biological characteristic with the lymphoid variants of BCR-ABL(+) leukemia (i.e. BCR-ABL acute lymphoblastic leukemia and the acute lymphoblastic variant of CML): deletions of the antigen receptor genes IGH, TCR, IKZF1, and CDKN1A/B. This phenomenon has never been observed in CML (11, 14).

The advent of tyrosine kinase inhibitors (TKIs) has significantly improved the prognosis for patients with CML and Ph+ ALL. These agents are also now used to treat patients with BCR-ABL(+) AML, but there is still no consensus on specific dosing regimens, the duration of therapy or combination protocols with standard chemotherapy. Previous studies have reported favorable hematological responses in BCR-ABL(+) AML patients treated with imatinib (3, 15).According to documented case reports, imatinib is usually given as an initial daily dose of 400 mg in combination with chemotherapy. Some patients continue to receive imatinib for over one year, achieving and maintaining sustained disease remission (16, 17).Gondran et al. treated patients with a combination of chemotherapy and imatinib. They found that this treatment significantly improved CR and OS rates, and reduced recurrence rates. They also demonstrated that sequential HSCT prolongs both recurrence-free survival (RFS) and OS (18).Unlike CML, the pathogenesis of AML is more complex and highly heterogeneous. The current treatment for BCR-ABL(+) AML primarily involves standard AML chemotherapy regimens combined with TKI drugs. Later-stage sequential HSCT is an effective treatment option that improves patients’ prognosis.

A previous French study reported an overall survival rate of 68.0% for BCR-ABL(+) AML patients undergoing HSCT over a period of two years (19),A survey by the European Society for Blood and Marrow Transplantation found that, following transplantation, patients had an overall survival rate of 54% and a complete remission rate of 84% after five years (20).A cohort study evaluating the long-term prognosis of BCR-ABL(+) AML patients found that a combination of chemotherapy and imatinib achieved a CR rate of approximately 75.9%. Among the 17 patients who subsequently underwent HSCT bridging, the five-year overall survival rate was 69.3%. In contrast, the median OS for the 12 patients who did not receive HSCT was only 6.25 months. These comparative findings demonstrate the efficacy of HSCT in achieving long-term disease control in patients with BCR-ABL(+) AML (21).V. Lazarevic et al. evaluated the efficacy of HSCT in patients with BCR-ABL(+) AML, enrolling 57 subjects. Seventy percent of patients had received TKI therapy prior to transplantation, achieving a CR1 rate of 79 percent. At the time of HSCT, 36.1% (14/40) of patients were MRD-negative. Following HSCT, 16 out of 26 (61.5%) MRD-positive patients achieved MRD negativity. At a median follow-up period of 6.3 years, the 5-year LFS and OS rates were 44.2% and 53.8%, respectively. Among CR1 patients, the 5-year OS rate approached 60%. Pre-transplant TKI use was associated with 5-year OS rates of 60% versus 34%. Conversely, the study found that HSCT increased the rate of deep molecular response observed pre-transplant from 34% to 63.4% (20).Min et al. reported that all seven MRD-negative patients survived after HSCT. In contrast, the five-year overall survival rate for the remaining ten MRD-positive patients prior to HSCT was only 44.4% (21).This suggests that combining TKIs with standard AML chemotherapy produces additive or synergistic effects, and that HSCT improves the prognosis of BCR-ABL(+) AML patients. Therefore, it is reasonable to conclude that the optimal treatment strategy for BCR-ABL(+) AML patients is to achieve deep molecular remission as early as possible, followed by sequential HSCT. Patients with persistent BCR-ABL fusion gene or MRD positivity should particularly be scheduled for Allo-HSCT at the earliest opportunity.

There is still no unified consensus on TKI regimens for patients after transplantation. Sun et al. reported on a 19-year-old female patient who resumed imatinib therapy at a dose of 300 mg/day, 74 days after HSCT and continued to take it for 15 months post-HSCT. The patient remained in CR 48 months after diagnosis, and the authors recommended at least one year of imatinib maintenance therapy following HSCT (22).A recent report by the European Society for Blood and Marrow Transplantation (EBMT) found that 18 patients received TKIs after transplantation for maintenance or relapse therapy. However, the sample size was too small to draw conclusions about the effectiveness of these drugs in treating BCR-ABL(+) AML (20).

The diagnosis of post-cytotoxic therapy acute myeloid leukemia (AML-pCT) requires the fulfillment of two key criteria: it must meet the WHO diagnostic criteria for AML, and a history of prior chemotherapy or radiotherapy must be confirmed. Epidemiological data indicate that AML-pCT accounts for 5–10% of adult AML cases and, compared to primary AML, exhibits a higher incidence of cytogenetic abnormalities. Common genetic mutation types include TP53, PPM1D, FLT3, TET2, DNMT3A and IDH. Specific genetic mutations and cytogenetic abnormalities are associated with exposure to various cytotoxic agents. Major cytotoxic therapies include alkylating agents, topoisomerase II inhibitors, antimetabolites, radiation therapy and immunomodulators. AML-pCT most commonly occurs 2–3 years after topoisomerase II inhibitor treatment, 5–7 years after treatment with alkylating agents or radiation therapy, and within months or years of immunosuppressant use. In terms of prognosis, AML-pCT has a poor outlook, with a median OS of only 8–14 months following standard chemotherapy (23, 24).

This patient has a history of malignant chest wall tumors spanning more than 10 years but is now in remission; PET-CT scans show no evidence of tumor cell activity, and it has been difficult to trace the specific drugs and dosages used in previous radiotherapy and chemotherapy. This patient had no prior history of CML. Peripheral blood and bone marrow smears revealed a blast cell count of over 20%. Bone marrow smears revealed no increase in mid- or late-stage myelocytes or basophils, no signs of dysproliferative hematopoiesis, no splenomegaly and no specific cytogenetic abnormalities. The diagnosis was BCR-ABL(+) AML with concomitant adverse prognostic genes. Although the relationship with AML-pCT remains unclear at this time, HSCT is the treatment of choice for improving patient outcomes.

ASXL1 plays a decisive role in maintaining genomic and expression stability across different gene loci. It is associated with the early stages of leukemia development and has been identified by the European Leukemia Network as a high-risk genetic predictor. Meta-analysis results indicate that ASXL1 significantly reduces survival in AML patients (25).Paschka et al. analyzed the prognosis of AL patients with ASXL1 mutations and found that, compared to patients without ASXL1 mutations, those with mutations exhibited significantly lower CR rates and five-year OS rates (26).RUNX1 acts as the ‘master switch’ for hematopoietic differentiation, controlling the maturation of myeloid progenitor cells into mature blood cells. When RUNX1 mutations cause differentiation arrest and the inactivation of tumor suppressor pathways, leukemia cells acquire ‘stem-like’ properties. Jalili et al. conducted a meta-analysis which concluded that RUNX1 mutations are associated with a poor prognosis, resistance to induction therapy and reduced patient survival rates (27).Experiments on mouse models showed that mutations in both RUNX1 and ASXL1 at the same time promote cellular renewal in the bone marrow and speed up the transformation of leukemia (28).Earlier studies reported that the ASXL1/RUNX1 mutation genotype has an additive adverse effect on genetics, with a significant interaction between the two mutations. Patients with the ASXL1/RUNX1 genotype were found to have a mortality risk nearly twice that of those lacking this dual mutation (26).The unique biological characteristics of BCR-ABL(+) AML currently prompt all centers to recommend a combination of chemotherapy and TKI for induction remission, with the aim of achieving deeper biological responses as early as possible. This is followed by HSCT to improve patients’ quality of life. The patient therefore received chemotherapy combined with TKI therapy. After one cycle of treatment, CR was rapidly achieved with MRD negativity. However, BCR-ABL remained positive throughout, indicating a high risk of relapse. Consequently, Allo-HSCT was performed. As expected, the patient subsequently underwent hematopoietic reconstitution, with follow-up testing showing BCR-ABL negativity. Following the transplant, the patient continued to take imatinib orally. According to previous literature reports and PH+ALL transplant guidelines, if BCR-ABL remains negative during continuous monitoring throughout treatment, TKI therapy should be maintained for at least one year. If negativity is not sustained, or if BCR-ABL becomes positive again after turning negative, ABL kinase domain mutation testing should be performed to determine whether to switch TKIs. This patient achieved disease remission with negative BCR-ABL gene status at follow-up after HSCT. This further validates the potential therapeutic advantage of this regimen for BCR-ABL(+) AML.

BCR-ABL(+) AML is a relatively rare subtype that exhibits distinct clinical characteristics and high heterogeneity, making it a poor-prognosis subtype. This case study suggests that combining TKI drugs with standard chemotherapy regimens can improve clinical outcomes. Sequential HSCT can further improve disease remission, thereby enhancing patient prognosis and quality of life. Using TKI drugs after transplantation may delay disease recurrence. However, this study is only a case report with a relatively short follow-up period. Therefore, future studies with larger sample sizes and longer follow-up periods are needed to validate these findings.

## Data Availability

The original contributions presented in the study are included in the article/Supplementary Material. Further inquiries can be directed to the corresponding author.
